# A prospective cohort study of clinical characteristics and outcomes in Chinese patients with estrogen receptor-negative/progesterone receptor-positive early breast cancer

**DOI:** 10.1007/s10549-023-06964-6

**Published:** 2023-05-18

**Authors:** Yu Fan, Xiaorong Zhong, Yu Wang, Zhu Wang, Ting Luo, Yanping Wang, Hong Zheng

**Affiliations:** Breast Center and Multi-omics Laboratory of Breast Diseases, West China Hospital, Sichuan University, Chengdu, 610041 People’s Republic of China

**Keywords:** Breast cancer, ER-negative/PR-positive, Mortality, Locoregional recurrence, Distant recurrence

## Abstract

**Purpose:**

This study aimed to examine the clinical characteristics and outcomes of patients with estrogen receptor-negative (ER−)/progesterone receptor-positive (PR+) early breast cancer. We also aimed to investigate the benefits of adjuvant endocrine therapy (ET) in this patient population.

**Methods:**

Patients with early breast cancer diagnosed at West China Hospital were divided into the ER−/PR+, ER+, and ER−/PR− groups. The chi-square test was used to analyze differences in clinical and pathological features among the groups. Multivariable Cox and Fine–Gray regression models were used to compare mortality and locoregional recurrence (LRR)/distant recurrence (DR), respectively. We performed a subgroup analysis to determine which ER−/PR+ patients can benefit more from ET.

**Results:**

From 2008 to 2020, we enrolled 443, 7104, and 2892 patients into the ER−/PR+, ER+, and ER−/PR− groups, respectively. The ER−/PR+ group showed more unfavorable clinical features and aggressive pathological characteristics than the ER+ group. The mortality, LRR, and DR rates were higher in the ER−/PR+ than in the ER+ group. Most clinical features and pathological characteristics were similar between the ER−/PR+ and ER−/PR− group and their outcomes were comparable. In the ER−/PR+ group, patients who received ET showed significantly lower LRR and mortality rates than those who did not; however, no difference was observed in DR. Subgroup analysis suggested that ER−/PR+ patients age ≥ 55 years, and postmenopausal status can benefit from ET.

**Conclusion:**

ER−/PR+ tumors have more aggressive pathological characteristics and more unfavorable clinical features than ER+ tumors. ET can reduce the LRR and mortality rates in ER−/PR+ patients. Postmenopausal and age ≥ 55 years ER−/PR+ patients can benefit from ET.

**Supplementary Information:**

The online version contains supplementary material available at 10.1007/s10549-023-06964-6.

## Introduction

For patients with early-stage breast cancer (BC) with positive hormone receptor status, 5–10 years of adjuvant endocrine therapy (ET) can significantly reduce the recurrence and mortality rates. Immunohistochemistry (IHC) testing for hormone receptor status is recommended for patients with newly diagnosed primary or metastatic BC [1].

Patients with estrogen receptor (ER)-positive BC (ER expression 1–100%) are known to benefit from ET. Patients with ER-negative/progesterone receptor (PR)-positive BC may be considered for ET; however, limited data are available for this patient group because ER−/PR+ BC accounts for < 10% of all BC cases [2–4]. Owing to the rarity of this subtype, few studies have assessed the response to ET in ER−/PR+ patients, and many prospective studies excluded this patient population [5].

Some studies have suggested that the ER−/PR+ subtype is biologically implausible given the co-expression pathway of ER and PR in BC [6, 7]. Other studies have indicated that most ER−/PR+ BC cases may represent false-negative IHC results for ER [8]. However, some studies have also reported that the mechanism of positive PR expression in ER- cases may be explained by the predominance of a variant form of ER [9, 10], the presence of ER missense mutations [11], or the activation of an alternative pathway [12]. Additionally, a study of ER−/PR+ BC cell lines demonstrated that PR can be expressed independently of the regulatory mechanisms of ER [13]. Thus, ER−/PR+ BC may represent a rare biological entity [14].

Whether ER−/PR+ patients can benefit from ET is highly controversial. A previous study indicated that patients with ER−/PR+ BC could benefit from tamoxifen therapy [15]. Another study concluded that adjuvant tamoxifen therapy might not provide a survival benefit for patients with high-grade ER−/PR+ tumors but was recommended for patients with low-grade ER−/PR+ tumors [16]. Conversely, a meta-analysis showed that only ER status, not PR status, was statistically significantly associated with tamoxifen response [5]. Another study reported that patients with ER−/PR+ BC who received ET had shorter survival times than those who did not [17].

In this study, we investigated a prospective cohort of 10,439 patients with early-stage BC diagnosed at West China Hospital (WCH) between 2008 and 2020. We compared the clinical-pathological features and survival outcomes of ER−/PR+ patients with those of ER+ and ER−/PR− patients. We also investigate the benefits of ET in the ER−/PR+ patient population.

## Methods

### Study design

**Fig. 1:**
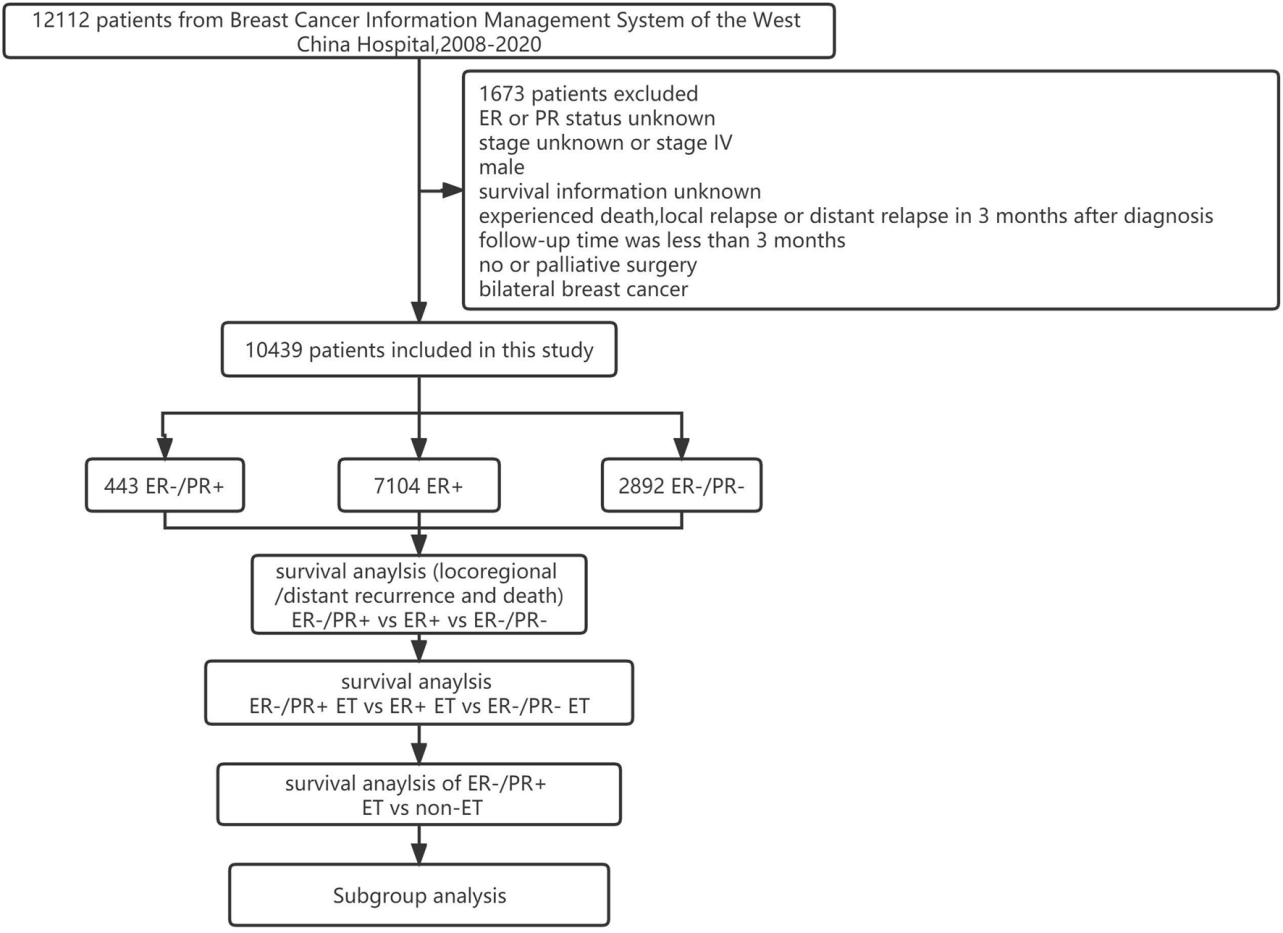
A flowchart of the study design and patient selection

A flowchart of the study design and patient selection process is shown in Fig. 1.

### Study population

Since 2008, patients with BC have been prospectively enrolled in the Breast Cancer Information Management System (BCIMS) of the WCH of Sichuan University [18]. Physicians collected medical records, pathological diagnosis information, and treatment data. Outpatient or telephone follow-up was performed every 3–4 months for the first 2 years, every 6 months for the next 3–5 years, and every year thereafter. This study was approved by the Clinical Test and Biomedical Ethics Committee of WCH, Sichuan University (reference no. 2012-130). All patients provided written informed consent. From 2008 to 2020, a total of 12,112 patients were registered in the BCIMS. Patients with no ER or PR status information, male sex, no survival information, experienced early events within 3 months or had < 3 months of follow-up, stage IV disease or no stage information, no or palliative surgery, or bilateral BC were excluded. Finally, 10,439 patients were included in this study.

Data on demographic features (age, residence, educational level, menopausal status, and body mass index [BMI]), clinical characteristics (human epidermal growth factor receptor 2 [HER2] status, Ki67 expression, CK5/6 status, tumor-node-metastasis [TNM] stage, histological type, and grade), and treatment modes (ET, chemotherapy, and radiotherapy) were collected.

### Pathological diagnosis and IHC

All pathological evaluations and IHC tests were performed at our hospital. Using antibodies selected by our institution, IHC was performed by staining for ER, PR, and HER2 on paraffin-embedded slides after deparaffinization, rehydration, and antigen retrieval [19]. ER or PR status was defined according to the percentage of tumor cells that positively expressed ER or PR; tumors with ≥ 1% stained cells nuclei were considered positive. The staining intensity of ER or PR was not included in our study. HER2 status was initially assessed using IHC and scored using a semi-quantitative scoring system. The status was confirmed using fluorescence in situ hybridization (FISH) in IHC equivocal cases (score 2+) according to the 2007 American Society of Clinical Oncology/College of American Pathologists guidelines [20]. HER2 IHC 0–1+ or FISH-negative tumors were considered negative; IHC 3+ or FISH-positive tumors were considered positive; and IHC 2+ tumors without FISH results were considered to have an uncertain status.

### *ESR1* mRNA expression

*ESR1* mRNA expression was detected using next-generation sequencing in 14 ER−/PR+ and 128 ER+ patients. RNA sequencing of frozen tumor specimens was performed on the Illumina Novaseq S6000 platform, as previously described. After quality control, the readings were mapped to the reference genome using HISAT2 version 2.0.5 [21]. The fragments per kilobase of exon per million mapped fragments (FPKM) values of *ESR1*, representing ER mRNA expression, were calculated according to a previously described method [22]. This part of the study was separately approved by the Clinical Test and Biomedical Ethics Committee of WCH, Sichuan University (reference no. 2019-16).

### Outcome assessment and statistical analysis

Mortality, locoregional recurrence (LRR), and distant recurrence (DR) were defined as death from any cause, tumor recurrence in the ipsilateral chest wall or regional lymph nodes, and disease recurrence in distant organs, respectively.

The patients were classified into three groups: ER−/PR+, ER+, and ER−/PR−. The chi-square test was used to compare the demographic features, clinical characteristics, and treatment modes among the three groups. The *t*-test was used to compare intergroup differences between two continuous variables, and two-way analysis of variance was used to compare intergroup differences among three or more continuous variables. A univariate analysis was performed to determine which covariates to adjust for in a multivariable analysis using Cox proportional hazard or Fine–Gray competing risk regression models. Mortality incidence curves were constructed using Kaplan–Meier survival analysis, and differences between groups were compared using the log-rank test. The Fine–Gray competing risk regression was used to compare the LRR and DR rates between groups. Death from any cause was considered a competing risk event for LRR and DR. R version 4.1.0 was used for statistical analysis. A two-tailed *P* value of < 0.05 was considered statistically significant.

## Results

### Proportion of ER−/PR+ patients

Among the 10,439 patients included in this study, 443 (4.25%) were ER−/PR+, 7104 (68.05%) were ER+, and 2892 (27.7%) were ER−/PR−. The last follow-up date was November 2021. The IHC stain of ER and PR in the ER−/PR+ group was shown in supplemental Fig. 1. We reviewed the proportion of ER−/PR+ patients every year during the study period. The proportion of ER−/PR+ patients varied from 1.41% in 2009 to 9.68% in 2014 (Supplemental Fig. 2a). We also compared the *ESR1* mRNA expression levels between ER−/PR+ and ER+ patients. We observed that the *ESR1* mRNA expression levels in 14 ER−/PR+ patients were significantly lower than those in 128 ER+ patients (*P* = 1.3E−08) (Supplemental Fig. 2b), suggesting that the ER−/PR+ status cannot be explained by false-negative staining for ER. In addition, the percentage of PR-expressing cells was significantly lower in ER−/PR+ tumors than in ER+/PR+ tumors. In 82.84% of ER−/PR+ tumors, 1–20% of cells expressed PR, indicating that most of the tumor cells may be ER−/PR−. Moreover, the tumors in this group had a high degree of intra-tissue heterogeneity (Supplemental Fig. 2c).

### Demographic features, clinicopathological characteristics, and treatment modes

Table 1 shows that ER−/PR+ patients were more frequently aged ≥ 55 years (31.2% vs. 24.9%) and more likely to be postmenopausal (51.5% vs. 41.1%) than ER+ patients. The ER−/PR+ group also had a higher TNM stage (stage III, 34.8% vs. 27.5%), a higher proportion of HER2-positive patients (43.8% vs. 19.9%), higher Ki67 expression (Ki67 ≥ 30%) (77.8% vs. 50.7%), and a higher proportion of CK5/6-positive patients (45.5% vs. 5.8%) than the ER+ group. More importantly, the ER−/PR+ group had more patients with grade 3 tumors (78.6% vs. 44.8%) than the ER+ group. Fewer patients received ET (63.2% vs. 91%), but more patients received chemotherapy (96.6% vs. 92.8%) in the ER−/PR+ group than in the ER+ group.

**Table 1:** Characteristics of patients with ER−/PR+, ER+ or ER−/PR− BC

	ER−/PR+ (*N* = 443)	ER+ (*N* = 7104)	ER−/PR− (*N* = 2892)	*P* ER−/PR+ Vs ER+	*P* ER−/PR+ Vs ER−/PR−
Age					
< 40	67 (15.1%)	1235 (17.4%)	444 (15.4%)	0.011	0.939
40–54	238 (53.7%)	4102 (57.7%)	1571 (54.3%)		
≥ 55	138 (31.2%)	1767 (24.9%)	877 (30.3%)		
Education (years)					
0–6	87 (20%)	1169 (16.7%)	550 (19.3%)	0.083	0.738
7–12	241 (55.5%)	3854 (55%)	1640 (57.5%)		
> 12	106 (24.4%)	1985 (28.3%)	662 (23.2%)		
BMI (kg/m^2^)					
< 24	303 (68.9%)	4554 (64.6%)	1843 (64.5%)	0.076	0.083
≥ 24	137 (31.1%)	2497 (35.4%)	1014 (35.5%)		
Residence					
Rural	129 (29.1%)	1845 (26%)	764 (26.4%)	0.164	0.255
Urban	314 (70.9%)	5250 (74%)	2128 (73.6%)		
Menopausal status					
Pre	215 (48.5%)	4182 (58.9%)	1445 (50%)	< 0.0001	0.609
Post	228 (51.5%)	2922 (41.1%)	1447 (50%)		
TNM stage					
0	2 (0.5%)	92 (1.3%)	48 (1.7%)	0.004	0.023
I	85 (19.2%)	1583 (22.3%)	629 (21.7%)		
II	202 (45.6%)	3478 (49%)	1376 (47.6%)		
III	154 (34.8%)	1951 (27.5%)	839 (29%)		
HER2 status					
Negative	208 (47%)	4725 (66.5%)	1387 (48%)	< 0.0001	0.922
Uncertain	41 (9.3%)	968 (13.6%)	260 (9%)		
Positive	194 (43.8%)	1411 (19.9%)	1245 (43%)		
Ki67					
< 30%	96 (22.2%)	3407 (49.3%)	597 (21.4%)	< 0.0001	0.765
≥ 30%	337 (77.8%)	3507 (50.7%)	2192 (78.6%)		
CK5/6					
Negative	205 (54.5%)	5789 (94.2%)	1338 (55.6%)	< 0.0001	0.741
Positive	171 (45.5%)	359 (5.8%)	1069 (44.4%)		
Grade					
1–2	71 (21.4%)	3269 (55.2%)	369 (16.4%)	< 0.0001	0.031
3	261 (78.6%)	2652 (44.8%)	1875 (83.6%)		
Chemotherapy					
No	15 (3.4%)	511 (7.2%)	97 (3.4%)	0.003	1
Yes	428 (96.6%)	6593 (92.8%)	2795 (96.6%)		
Radiotherapy					
No	308 (69.5%)	4976 (70%)	2103 (72.7%)	0.858	0.18
Yes	135 (30.5%)	2128 (30%)	789 (27.3%)		
ET					
No	163 (36.8%)	638 (9%)	2680 (92.7%)	< 0.0001	< 0.0001
Yes	280 (63.2%)	6466 (91%)	212 (7.3%)		

ER−/PR− and ER−/PR+ patients showed similar age, educational characteristics, menopausal status, HER2 status, Ki67% expression, and CK5/6 status; however, ER−/PR+ patients were more likely to have stage III disease (34.8% vs. 29%). Moreover, the proportion of patients with grade 3 tumors was smaller in the ER−/PR+ group than in the ER−/PR− group (78.6% vs. 83.6%). In terms of treatment mode, more ER−/PR+ patients than ER−/PR− patients received ET (63.2% vs. 7.3%) (Table 1). These results indicated that ER−/PR+ tumors had more aggressive and unfavorable characteristics than ER+ tumors but showed similar characteristics to ER−/PR− tumors.

### Survival analysis of ER−/PR+, ER+, and ER−/PR− patients

**Fig. 2:**
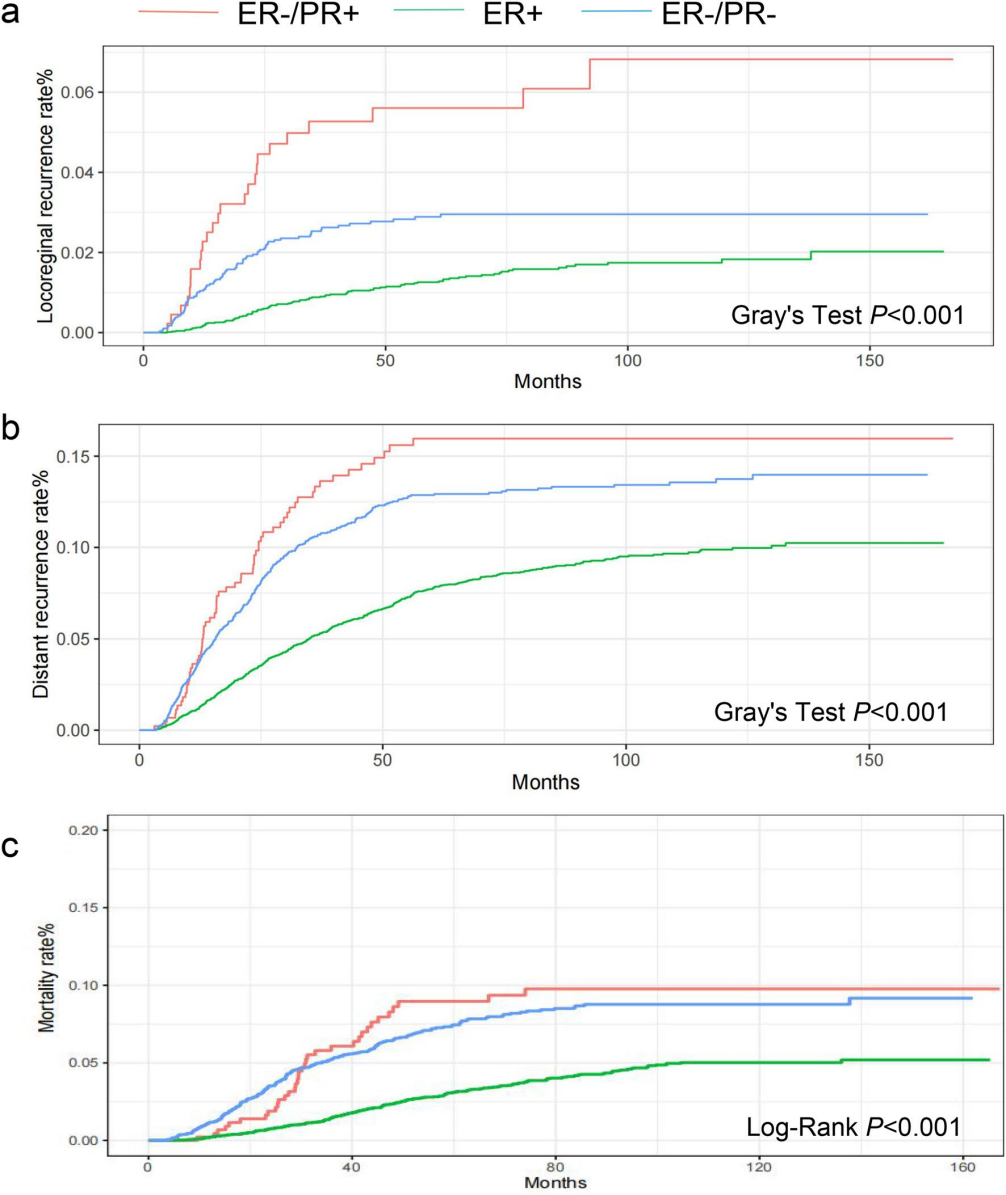
Outcome of the ER−/PR+, ER+ and ER−/PR− groups. **a** Cumulative incidence of LRR, **b** Cumulative incidence of DR, **c** Cumulative incidence of mortality in the ER−/PR+, ER+ and ER−/PR− groups. *ER* estrogen receptor, *PR* progesterone receptor, *LRR* locoregional recurrence, *DR* distant recurrence

The median follow-up time for the prospective cohort was 65.3 months. Of the patients, 472 died from any cause, 197 had LRR, 937 had DR, and 117 had both LRR and DR. The 5-year mortality rates were 9.0%, 3.1%, and 7.5% in the ER−/PR+, ER+, and ER−/PR− groups, respectively (log-rank *P* < 0.001). The 5-year LRR rates were 5.6%, 1.3%, and 2.9% in the ER−/PR+, ER+, and ER−/PR− groups, respectively (Gray’s test *P* < 0.001). The 5-year DR rates were 16.0%, 7.7%, and 13% in the ER−/PR+, ER+, and ER−/PR− groups, respectively (Gray’s test *P* < 0.001) (Table 2). ER+ patients presented a lower mortality risk (hazard ratio [HR] 0.41, 95% confidence interval [CI] 0.28–0.61, *P* = 7.5E−06), LRR risk (HR 0.26, 95% CI 0.15–0.44, *P* = 6.6E−07), and DR risk (HR 0.58, 95% CI 0.42–0.81, *P* = 0.001) than ER−/PR+ patients. However, no significant differences in mortality (HR 0.82, 95% CI 0.56–1.2, *P* = 0.3) and DR (HR 0.88, 95% CI 0.63–1.24, *P* = 0.77) were observed between the ER−/PR− and ER−/PR+ groups. Surprisingly, the LRR risk was significantly lower (HR 0.43, 95% CI 0.23–0.8, *P* = 0.007) in the ER−/PR− group than in the ER−/PR+ group. In multivariable Cox regression and Fine–Gray competing risk regression analyses, HRs and *P* values were adjusted for covariates that had a *P* value of < 0.1 in the univariate analysis. The tumor grade was not adjusted because tumor grade information was not available for a considerable number of patients. (Fig. 2a–c, Table 3, and Supplemental Tables 1, 2, 3).

**Table 2:** 5-year incidence of mortality, LRR and DR among groups

	5-year mortality		5-year LRR		5-year DR	
	95%CI	Log-rank *P*	95%CI	Gray’s test *P*	95%CI	Gray’s test *P*
All patients		< 0.001		< 0.001		< 0.001
ER−/PR+	9% (6%, 11.9%)		5.6% (3.6%, 8.1%)		16% (12%, 20%)	
ER+	3.1% (2.6%, 3.5%)		1.3% (1.0%, 1.6%)		7.7% (7.1%, 8.4%)	
ER−/PR−	7.5% (6.4%, 8.5%)		2.9% (2.3%, 3.6%)		13% (12%, 14%)	
Patients received ET		0.001		< 0.001		< 0.001
ER−/PR+	6.1% (3%, 9%)		3.6% (1.9%, 6.4%)		16% (12%, 21%)	
ER+	2.7% (2.2%, 3.1%)		1.2% (0.93%, 1.5%)		7.5% (6.8%, 8.2%)	
ER−/PR−	6.4% (2.8%, 9.8%)		5.5% (2.9%, 9.2%)		16% (11%, 22%)	
ER−/PR+ patients		0.004		0.056		0.9
No ET	14.8% (8.1%, 21%)		9.5% (5.3%, 15%)		15% (9.7%, 22%)	
ET	6.1% (2.3%, 9%)		3.6% (1.9%, 6.4%)		16% (12%, 21%)

**Table 3:** The multivariate Cox regression analysis and Gray–Fine test for incidence outcomes among groups

	Mortality		LRR		DR	
	HR (95%CI)	*P*	HR (95%CI)	*P*	HR (95%CI)	*P*
All patients						
ER−/PR+	Reference		Reference		Reference	
ER+	0.41(0.28, 0.61)	7.50E−06	0.26 (0.15, 0.44)	6.60E−07	0.58 (0.42, 0.81)	0.001
ER−/PR−	0.82 (0.56, 1.2)	0.3	0.43 (0.23, 0.79)	0.007	0.88 (0.63, 1.24)	0.46
Patients received ET						
ER−/PR+	Reference		Reference		Reference	
ER+	0.51 (0.29, 0.91)	0.022	0.37 (0.19, 0.74)	0.005	0.65 (0.43,0.97)	0.037
ER−/PR−	1.2 (0.57, 2.5)	0.64	1.2 (0.51, 2.86)	0.67	1.29 (0.77, 2.14)	0.33
ER−/PR+ patients						
No ET	Reference		Reference		Reference	
ET	0.5(0.25, 0.98)	0.045	0.4(0.18,0.87)	0.02	1.21(0.71, 2.05)	0.49

### Survival analysis of ER−/PR+, ER+, and ER−/PR− patients treated with ET

**Fig. 3:**
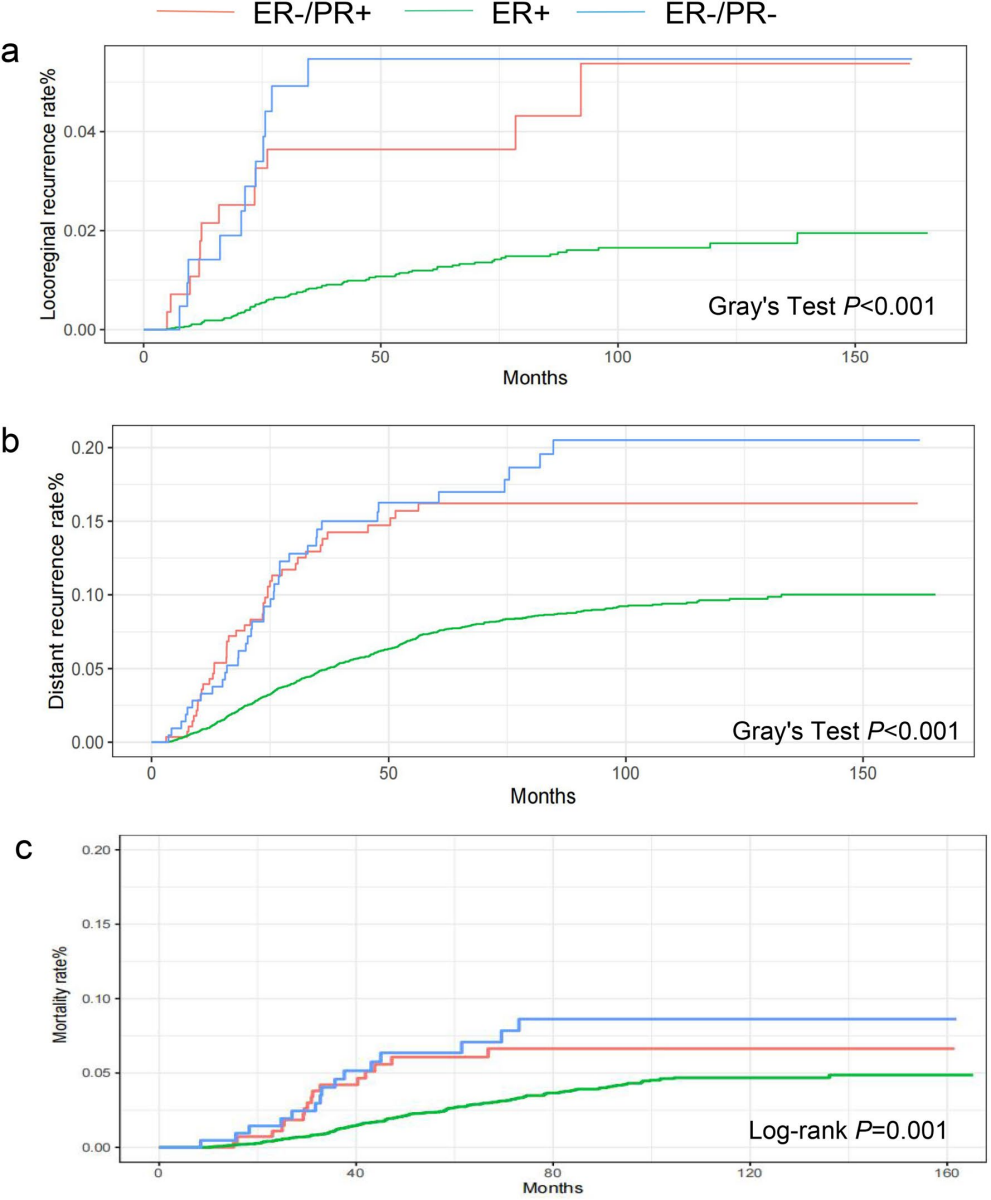
Outcome of the ER−/PR+, ER + and ER−/PR− groups who received ET. **a** Cumulative incidence of LRR, **b** Cumulative incidence of DR, **c** Cumulative incidence of mortality in the ER−/PR+, ER+ and ER−/PR− groups. *ET* adjuvant endocrine therapy

After ET, the ER+ group still showed better outcomes in terms of mortality, LRR, and DR than the ER−/PR+ group. However, no significant differences in mortality, LRR, and DR rates were observed between the ER−/PR+ and ER−/PR− groups. The 5-year mortality rates were 6.1%, 2.7%, and 6.4% in the ER−/PR+, ER+, and ER−/PR− groups, respectively (log-rank *P* = 0.001). The 5-year LRR rates were 3.6%, 1.2%, and 5.5% in the ER−/PR+, ER+, and ER−/PR− groups, respectively (Gray’s test *P* < 0.001). The 5-year DR rates were 16%, 7.5%, and 16% in the ER−/PR+, ER+, and ER−/PR− groups, respectively (Gray’s test *P* < 0.001) (Table 2). The ER+ group still presented a lower risk of mortality (HR 0.51, 95% CI 0.29–0.91, *P* = 0.022), LRR (HR 0.37, 95% CI 0.19–0.74, *P* = 0.005), and DR (HR 0.65, 95% CI 0.43–0.97, *P* = 0.037) than the ER−/PR+ group after ET. However, no differences in mortality (HR 1.2, 95% CI 0.57–2.5, *P* = 0.64), LRR (HR 1.2, 95% CI 0.51–2.86, *P* = 0.67), and DR (HR 1.29, 95% CI 0.77–2.14, *P* = 0.33) were observed between the ER−/PR+ and ER−/PR− groups after ET. In multivariable Cox regression and Fine–Gray competing risk regression analyses, HRs and *P* values were adjusted for covariates that had a *P* value of < 0.1 in the univariate analysis, except for tumor grade as mentioned above (Fig. 3a–c, Table 3, Supplemental Tables 4, 5, 6).

### Survival analysis of ER−/PR+ patients treated or not with ET and subgroup analysis

**Fig. 4:**
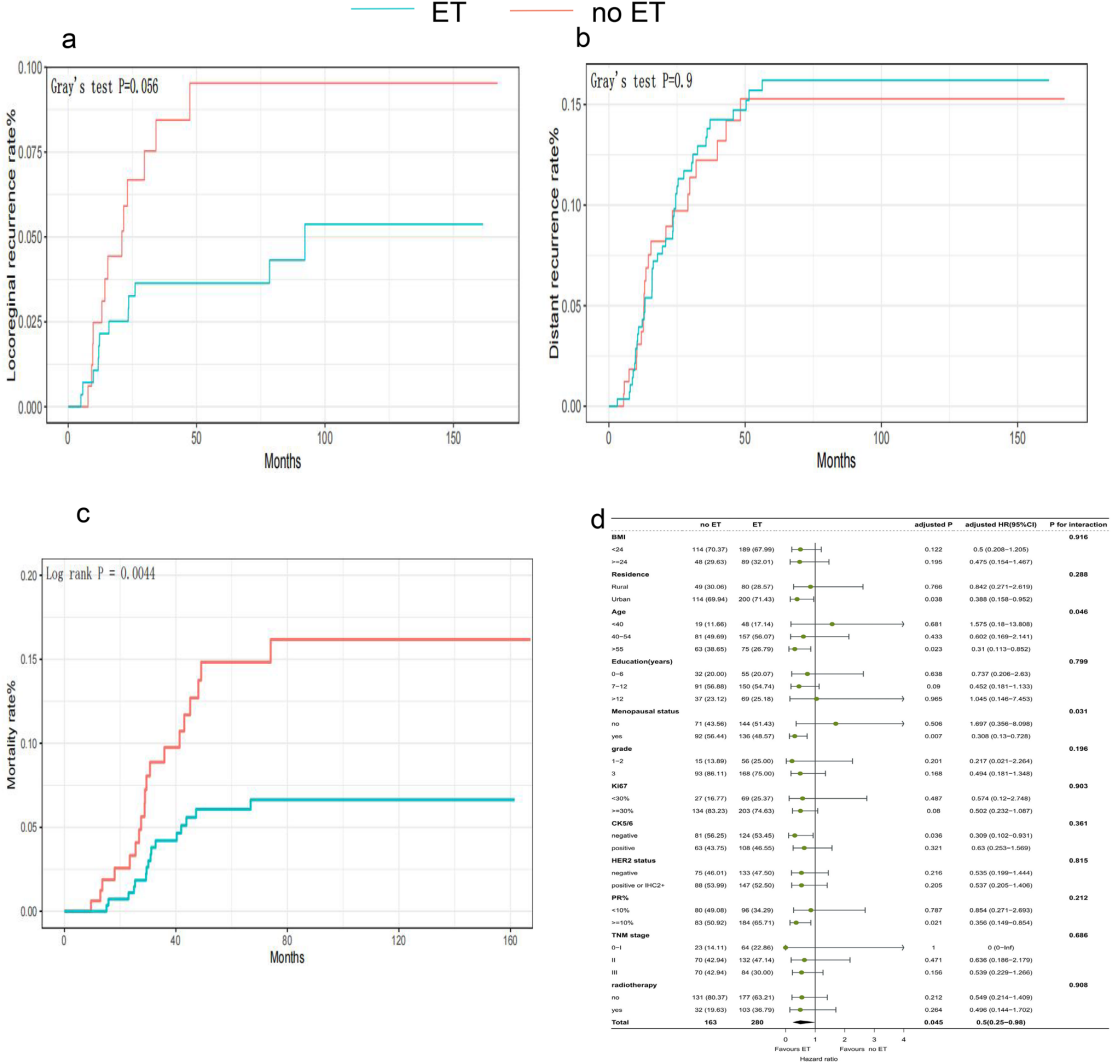
Outcome of the ER−/PR+ patients who received ET or not. **a** Cumulative incidence of LRR, **b** Cumulative incidence of DR, **c** Cumulative incidence of mortality by ER−/PR+ patients who received ET or not, **d** Subgroup analysis and interaction tests of mortality in ER−/PR+ patients who received ET or not

ET decreased the mortality rate but not the DR rate in the ER−/PR+ group. A decreased LRR risk was also observed in patients who received ET, with borderline significance by Gray’s test. The 5-year mortality rates were 14.8% and 6.1% in the no ET and ET groups, respectively (log-rank *P* = 0.004). The 5-year LRR rates were 9.5% and 3.6% in the no ET and ET groups, respectively (Gray’s test *P* = 0.056). The 5-year DR rates were 15% and 16% in the no ET and ET groups, respectively (Gray’s test *P* = 0.9) (Table 2). In ER−/PR+ patients, ET reduced the mortality risk by 50% (HR 0.5, 95% CI 0.25–0.98, *P* = 0.045) and the LRR risk by 60% (HR 0.4, 95% CI 0.18–0.87, *P* = 0.02) compared with the absence of ET. However, no difference was observed in DR (HR 1.21, 95% CI 0.71–2.05, *P* = 0.49). In the multivariable Cox regression and Fine–Gray competing risk regression analyses, HRs and *P* values were adjusted for covariates that had a *P* value of < 0.1 in the univariate analysis (Fig. 4a–c, Table 3, Supplemental Tables 7, 8, 9).

In addition, we performed a subgroup analysis to determine which subgroup of ER−/PR+ patients can gain a survival benefit from ET. Age, residence, education, menopausal status, and TNM stage had a *P* value of < 0.1 in the univariate analysis and were used as adjustment variables for the multivariable Cox regression analysis. Patients aged ≥ 55 years and postmenopausal patients gained a survival benefit from ET. Patients aged < 55 years and premenopausal patients did not appear to benefit from ET (for interaction, *P* values were 0.046 and 0.031, respectively) (Fig. 4d).

## Discussion

This prospective cohort study with a large sample size describes the survival rate of patients with ER−/PR+ early BC in WCH from 2008 to 2020. We found that the ER−/PR+ subtype was associated with more unfavorable clinical features and more aggressive pathological characteristics compared with ER+ BC but showed similar characteristics to ER−/PR− BC. The mortality, LRR, and DR rates of ER−/PR+ patients were significantly worse than those of ER+ patients, even after ET. Nevertheless, ET reduced the mortality and LRR risks, but not the DR risks, in ER−/PR+ patients. Furthermore, the mortality risk was significantly reduced by ET in patients with age ≥ 55 years, and postmenopausal status.

The proportion of ER−/PR+ patients in our database (4.24%) was higher than that in the Surveillance, Epidemiology, and End Results database (SEER) (1.6%) [23]. The difference between the two databases may be due to race because a retrospective study from South Korea showed that ER−/PR+ patients accounted for 9.4% of all patients with BC [24] and another study from China reported that the proportion of ER−/PR+ patients was 11% [25]. The proportion of PR−/ER+ patients in our study did not decrease with time, thereby excluding the possibility that the difference in proportion was caused by advances in IHC technology. In addition, the RNA sequencing data showed that the *ESR1* FPKM value was significantly lower in ER−/PR+ patients than in ER+ patients, suggesting that the classification of ER−/PR+ patients was not caused by false-negative staining for ER. We also observed that the PR expression percentage was significantly higher in ER+ patients than in ER−/PR+ patients, and > 80% of ER−/PR+ patients had PR expression between 1 and 20%. This indicates that other alternative mechanisms may be responsible for the expression of PR in ER−/PR+ patients. We noted that in 2013 WCH changed the ER and PR antibodies for pathological diagnosis, which may weaken the robustness of our results.

Similar to other studies, our results demonstrated that ER−/PR+ BC had more unfavorable clinical features and more aggressive biological characteristics, such as a higher stage, a higher tumor grade, a higher proportion of CK5/6-positive status [4], higher HER2 expression [26], and higher Ki67 expression [24, 27], than ER+ BC. Consistent with most studies [28, 29], our results also indicated that ER−/PR+ patients had a higher risk of mortality, LRR, and DR than ER+ patients, even after ET [25].

However, whether a survival difference exists between the ER−/PR+ and ER−/PR− groups remains controversial. Most studies indicated that ER−/PR+ patients have a better prognosis than ER−/PR− patients [16, 23, 30]. Other studies demonstrated no difference in survival between the ER−/PR+ and ER−/PR− groups [29]. Meanwhile, some studies demonstrated that ER−/PR+ patients have lower disease-specific survival rates than ER−/PR− patients [31]. We observed similar mortality and DR rates between the ER−/PR+ group and in the ER−/PR− group, although a higher LRR rate was observed in the ER−/PR+ patients. Several possible reasons can be suggested to explain these results. First, the expression percentage of PR was 1–20% in > 80% of ER−/PR+ patients, indicating that the tumor tissues predominantly consisted of ER−/PR− cells. Therefore, the prognosis may be similar between the ER−/PR+ and ER−/PR− groups. Second, in our cohort, ER−/PR+ patients had a higher TNM stage than ER−/PR− patients. There were more stage III patients and fewer stage 0–I patients in the ER−/PR+ group than in ER−/PR− group. Third, one-third of ER−/PR+ patients did not receive ET, which might have improved their survival rate.

Although the predictive value of ER in patients with early BC treated with ET is widely recognized, the predictive and prognostic significance of PR is still a topic of debate [32, 33]. Controversy remains about whether ET can improve the prognosis of ER−/PR+ patients. Dowsett et al. reported that PR+ patients could significantly benefit from tamoxifen treatment [34], and another study demonstrated that ET could improve relapse-free and overall survival [35]. However, a meta-analysis showed no benefit from tamoxifen therapy in patients with ER-poor BC, irrespective of the PR status [5]. Yang et al. suggested that in the ER−/PR+ group, only patients with low-grade tumors showed better overall and disease-free survival after ET [16]. Another study reported that ER−/PR+ patients who received ET had shorter survival times than those who did not [17].

In our cohort, ER−/PR+ patients who received ET showed significantly decreased mortality and LRR rates compared with those who did not receive ET; however, the DR rates did not differ between the two groups. A previous study reported that PR loss is more common than ER and HER2 loss in recurrent metastatic disease [36]. In other words, metastatic cell colonies may form mainly from ER−/PR− cells, which may explain why ET can significantly reduce local recurrence but not distant metastasis. A small number of ER−/PR− BC patients (7.3%) in our study cohort received ET. This may be due to inconsistent results between the core needle biopsy and the postoperative specimen or a second hormone receptor-positive BC.

Yamashita et al. reported that patients with an ER or PR expression percentage of ≥ 1% had better survival after relapse and suggested 1% as the cutoff value [37]. However, our subgroup analysis demonstrated that when the PR expression percentage was ≥ 10%, ET significantly reduced the risk of mortality in the ER−/PR+ group. The *P* for interaction was > 0.05, which may be due to the small number of ER−/PR+ patients. Therefore, this result may need to be confirmed in a study with a larger sample size. More randomized controlled studies are needed to decide the optimal PR threshold for making ET decisions. Because of the limited number of ER−/PR+ cases and some of patients’ HER2 status were equivocal, we did not stratify patients by HER2 status for survival analysis. Subgroup analysis suggested that HER2 status did not appear to alter the results of patients who used ET or not.

In addition, postmenopausal patients and those aged ≥ 55 years seemed to have significantly benefited from ET in terms of mortality compared with patients with a premenopausal status and age < 55 years. Our results provide a good basis for selecting ER−/PR+ patients for ET treatment.

## Conclusion

Our results demonstrated that ER−/PR+ tumors had more unfavorable clinical features and aggressive pathological characteristics than ER+ tumors. The prognosis in terms of mortality, LRR, and DR was worse in the ER−/PR+ group than in the ER+ group, even after ET. However, most clinical features and pathological characteristics were similar between the ER−/PR+ and ER−/PR− group and their outcomes were comparable. The LRR and mortality rates were reduced by ET in ER−/PR+ patients. Subgroup analysis suggested that patients age ≥ 55 years, and postmenopausal status can significantly benefit from ET.

## Supplementary Information

Below is the link to the electronic supplementary material.

Supplementary file1 (PDF 1417 kb)

Supplementary file2 (XLSX 22 kb)

## Data Availability

Data supporting the findings of this study are available upon request from the corresponding author.

## Abbreviations

ER: Estrogen receptor
PR: Progesterone receptor
ET: Adjuvant endocrine therapy
LRR: Locoregional recurrence
DR: Distant recurrence
BC: Breast cancer
IHC: Immunohistochemistry
BCIMS: Breast Cancer Information Management System
WCH: West China Hospital
BMI: Body mass index
HER2: Human epidermal growth factor receptor 2
TNM: Tumor-node-metastasis
FISH: Fluorescence in situ hybridization
FPKM: Fragments per kilobase of exon per million mapped fragments
HR: Hazard ratio
CI: Confidence interval
