# Supplementary material for: A prospective cohort study of clinical characteristics and outcomes in Chinese patients with estrogen receptor-negative/progesterone receptor-positive early breast cancer
Source: Breast Cancer Res Treat. 2023 May 18;200(2):171–82. doi: 10.1007/s10549-023-06964-6 (PMC10241679; doi:10.1007/s10549-023-06964-6)

Supplemental Fig. 1 Representative immunohistochemistry images and corresponding H&E images of breast tumors with ER-/PR+. a. ER-,PR20%+; b. ER-,PR70%+

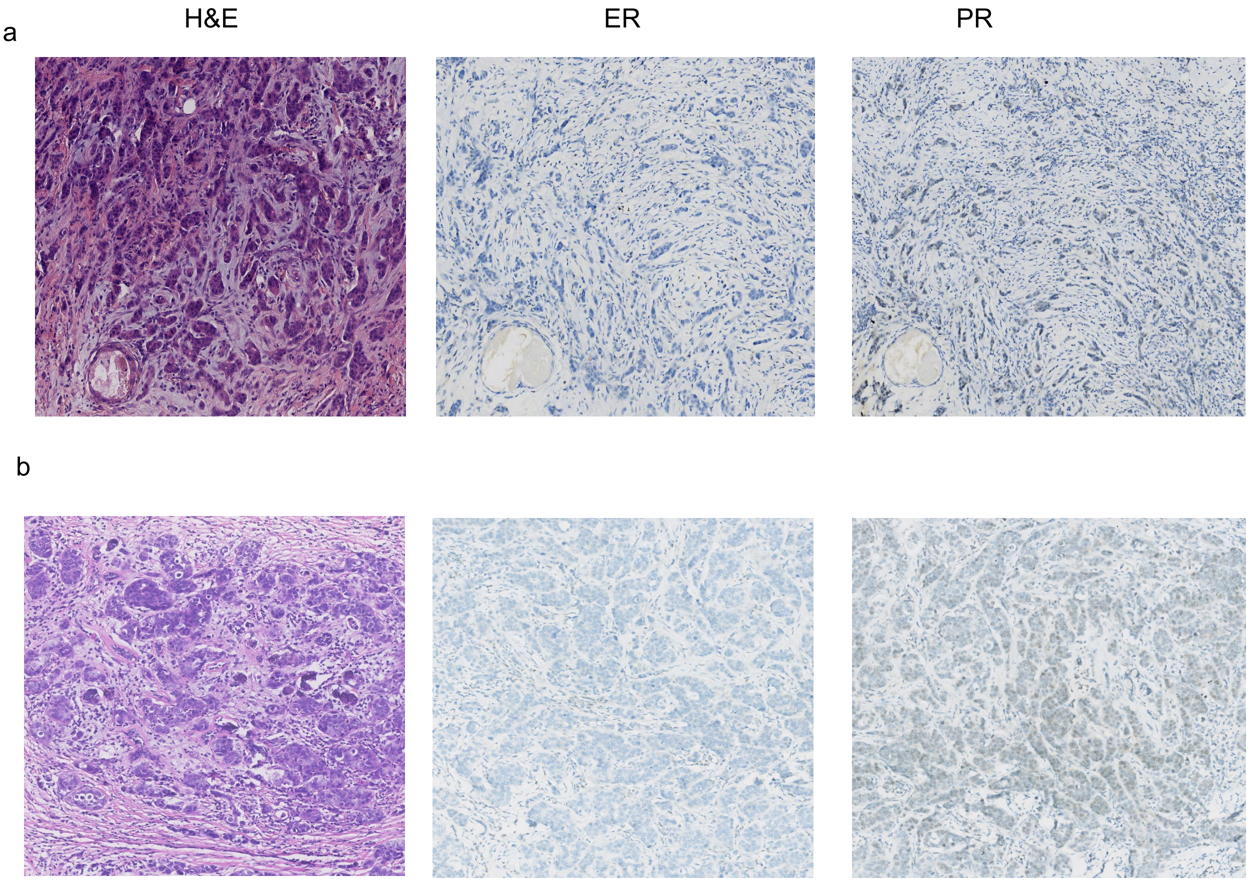

Supplemental Fig. 2 Percentage of patients by year and ER, PR expression. a. Percentage of ER-/PR+, ER+ and ER-/PR- patients by year. b. ESR1 mRNA expression in ER-/PR+ patients and ER+ patients. c. Percentage expression of PR in ER-/PR+ patients and ER+/PR + patients.

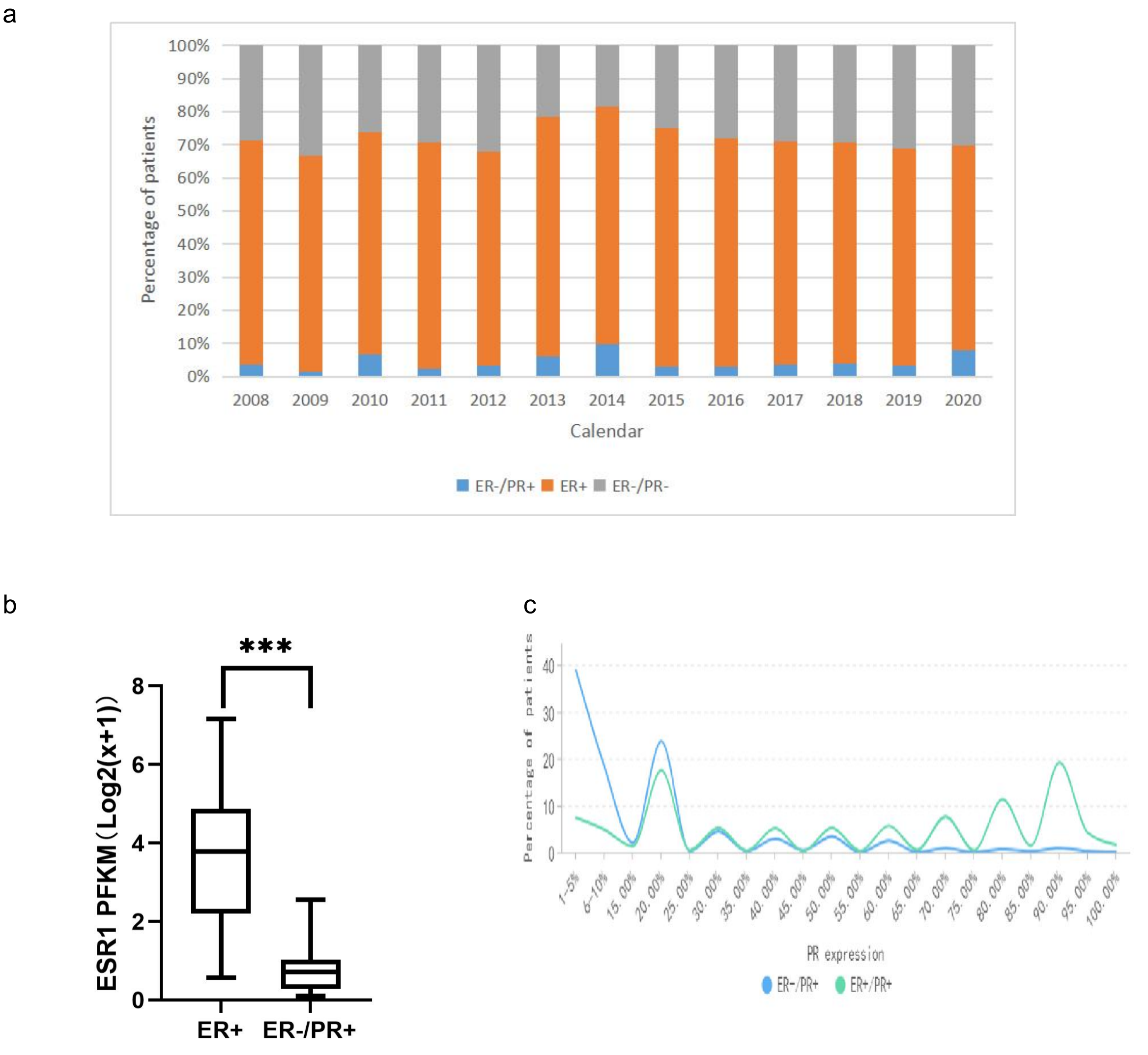

Supplement: Supplementary file 1 — Supplementary file1 (PDF 1417 kb) [file 10549_2023_6964_MOESM1_ESM.pdf]
